# Supplementary material for: Detection of Pancreatic Cancer miRNA with Biocompatible Nitrogen-Doped Graphene Quantum Dots
Source: Materials (Basel). 2022 Aug 20;15(16):5760. doi: 10.3390/ma15165760 (PMC9414703; doi:10.3390/ma15165760)
Supplement: Supplementary file 1 [file materials-15-05760-s001.zip › materials-1849000-supplementary.pdf]

materials

Supplementary Materials

Detection of Pancreatic Cancer miRNA with Biocompatible Nitrogen-Doped Graphene Quantum Dots

Ryan Ajgaonkar [1,2,†], Bong Lee [3,†], Alina Valimukhametova [3], Steven Nguyen [3], Roberto Gonzalez-Rodriguez [4], Jeffery Coffer [5], Giridhar R. Akkaraju [2] and Anton V. Naumov [3,*]

1 School of Medicine, University of Texas Rio Grande Valley, Edinburg, TX 78539, USA
2 Department of Biology, Texas Christian University, Fort Worth, TX 76129, USA
3 Department of Physics and Astronomy, Texas Christian University, Fort Worth, TX 76129, USA
4 Department of Physics, University of North Texas, Denton, TX 76203, USA
5 Department of Chemistry and Biochemistry, Texas Christian University, Fort Worth, TX 76129, USA
* Correspondence: a.naumov@tcu.edu; Tel.: +1-(713)-253-8775
† These authors contributed equally to this work.

Citation: Ajgaonkar, R.; Lee, B.; Valimukhametova, A.; Nguyen, S.; Gonzalez-Rodriguez, R.; Coffer, J.; Akkaraju, G.R.; Naumov, A.V. Detection of Pancreatic Cancer miRNA with Biocompatible Nitrogen-Doped Graphene Quantum Dots. *Materials* **2022**, *15*, 5760. https://doi.org/10.3390/ma15165760

Academic Editor: Daniela Iannazzo

Received: 19 July 2022
Accepted: 17 August 2022
Published: 20 August 2022

Publisher's Note: MDPI stays neu-tral with regard to jurisdictional claims in published maps and institu-tional affiliations.

Copyright: © 2022 by the authors. Licensee MDPI, Basel, Switzerland. This article is an open access article distributed under the terms and conditions of the Creative Commons Attribution (CC BY) license (https://creativecommons.org/licenses/by/4.0/).

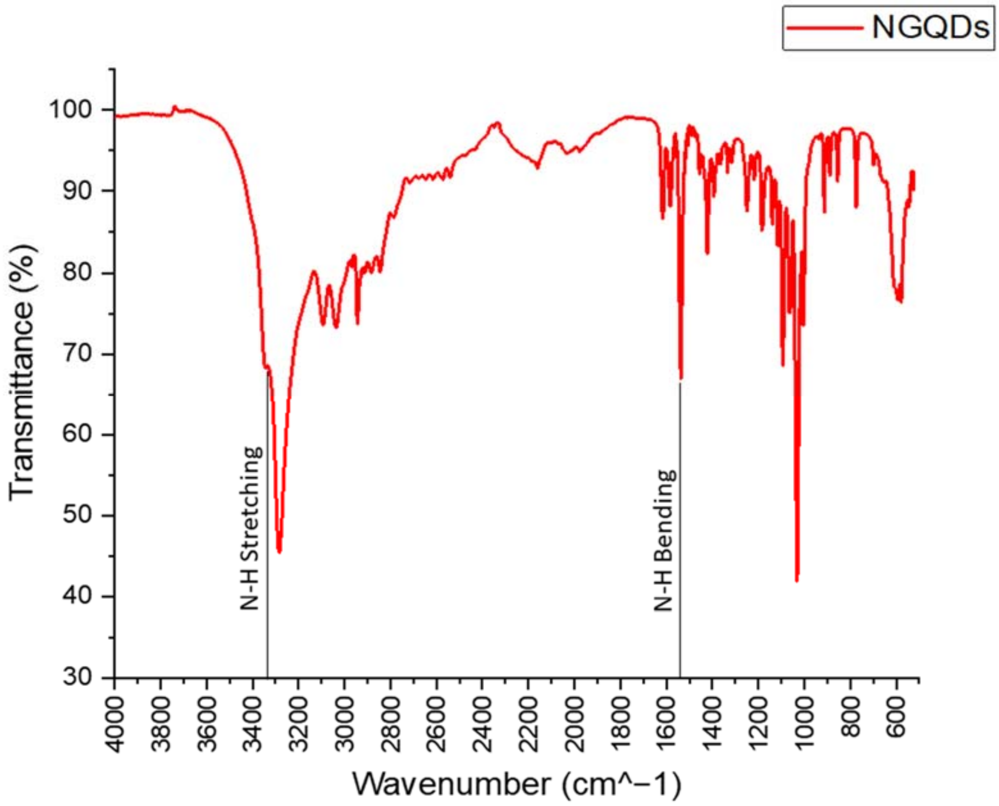

Figure S1. FTIR spectrum of NGQDs.

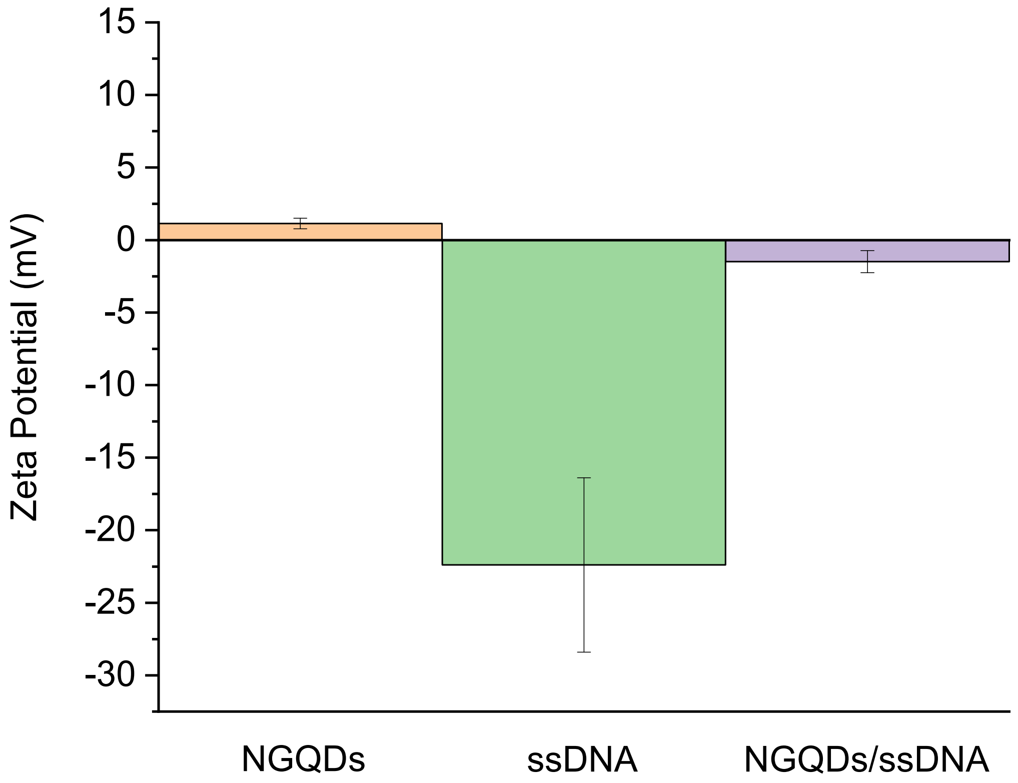

Figure S2. Zeta potential of NGQDs (orange), ssDNA (green), NGQDs/ssDNA (purple) with the corresponding values of 1.14 ± 0.36, -22.4 ± 6.00, and -1.48 ± 0.76 mV.

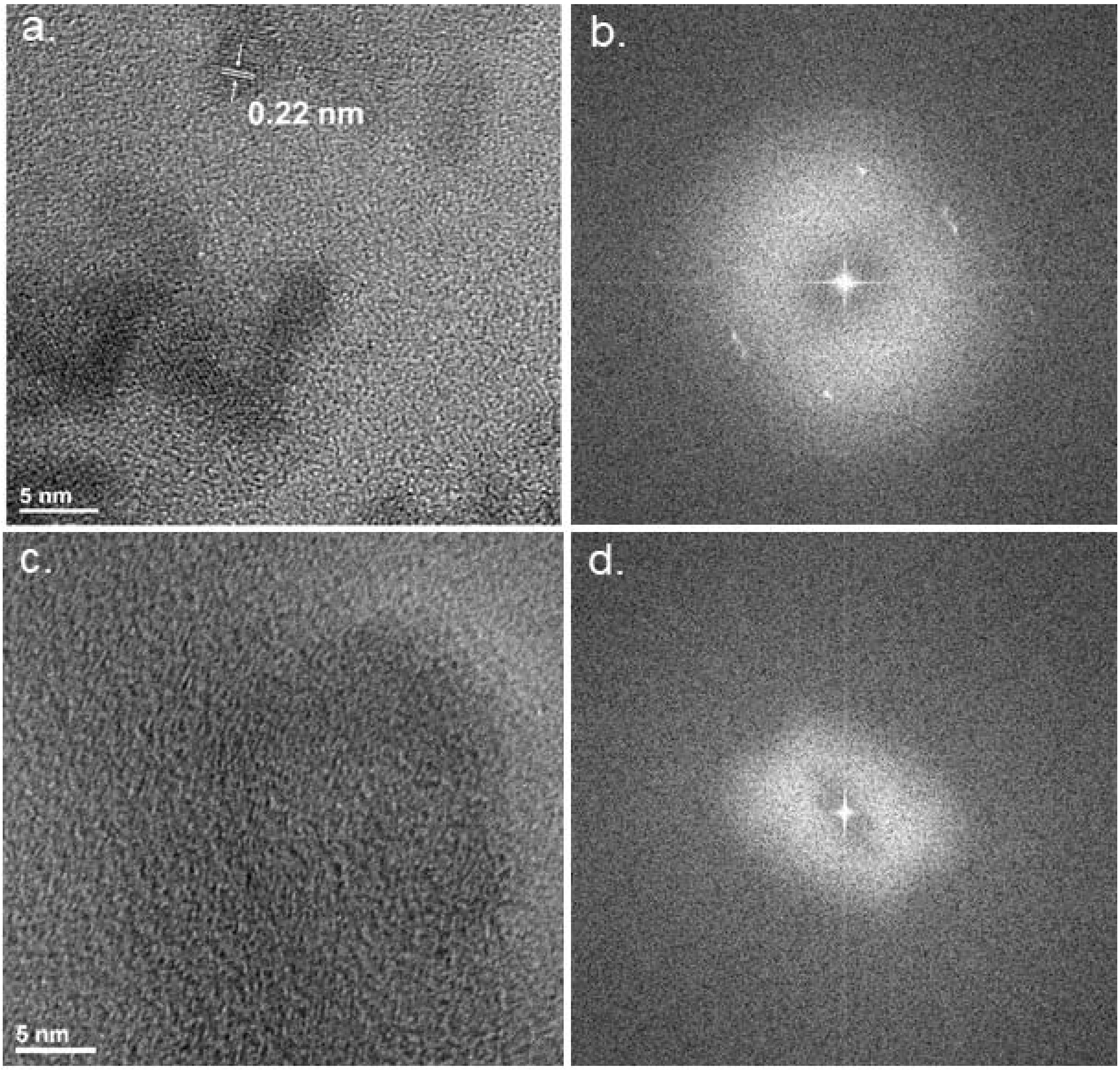

Figure S3. HRTEM and FFT of (a,b) NGQDs and (c,d) NGQDs/ssDNA.

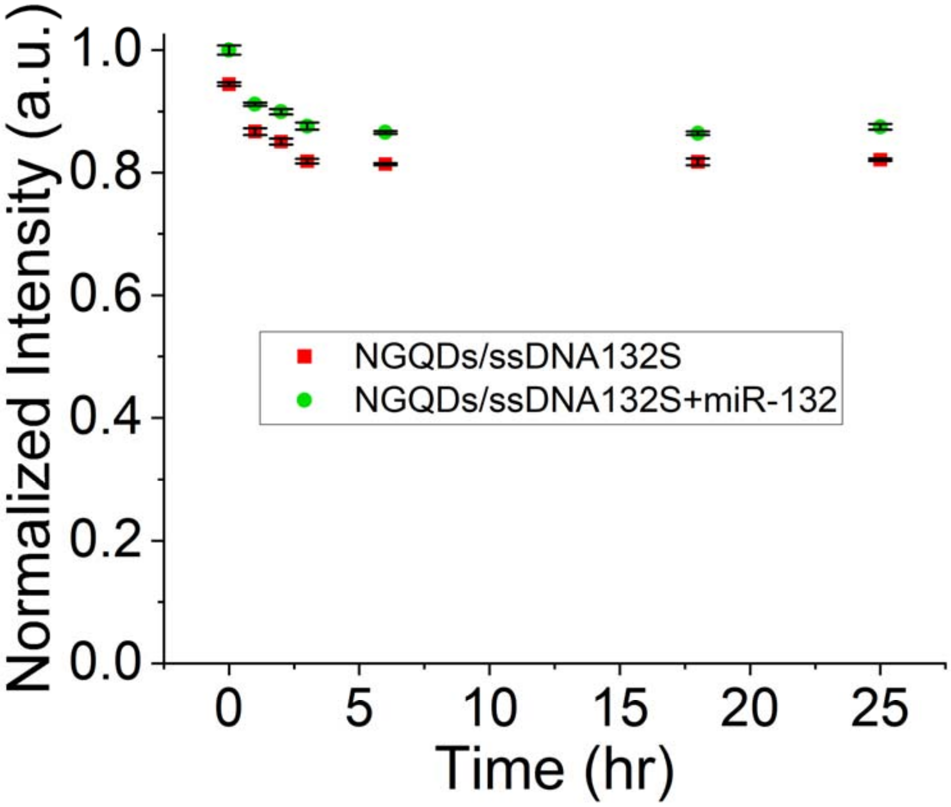

Figure S4. Stability test of NGQDs/ssDNA132S (red) and NGQDs/ssDNA132S+miR-132 (green).
